# Identifying Patients with Polycythemia Vera at Risk of Thrombosis after Hydroxyurea Initiation: The Polycythemia Vera—Advanced Integrated Models (PV-AIM) Project

**DOI:** 10.3390/biomedicines11071925

**Published:** 2023-07-07

**Authors:** Srdan Verstovsek, Ivan Krečak, Florian H. Heidel, Valerio De Stefano, Kenneth Bryan, Mike W. Zuurman, Michael Zaiac, Mara Morelli, Aoife Smyth, Santiago Redondo, Erwan Bigan, Michael Ruhl, Christoph Meier, Magali Beffy, Jean-Jacques Kiladjian

**Affiliations:** 1 Department of Leukemia, The University of Texas MD Anderson Cancer Center, Houston, TX 77030, USA; 2 Department of Internal Medicine, General Hospital of Sibenik-Knin County, 22000 Sibenik, Croatia; 3 Faculty of Medicine, University of Rijeka, 51000 Rijeka, Croatia; 4 Hematology, Oncology, Stem Cell Transplantation and Palliative Care, Internal Medicine C, University Medicine Greifswald, 17475 Greifswald, Germany; 5 Sezione di Ematologia, Dipartimento di Scienze Radiologiche ed Ematologiche, Università Cattolica, Fondazione Policlinico A. Gemelli IRCCS, 00168 Roma, Italy; 6 Novartis Ireland Limited, Dublin 4, D04 A9N6 Dublin, Ireland; 7 Novartis Pharma AG, CH-4056 Basel, Switzerland; 8 Novartis Farma SpA, 21040 Origgio, Italy; 9 Novartis Pharmaceuticals UK Limited, London W12 7FQ, UK; 10 Novartis Farmaceutica, S.A., 28033 Madrid, Spain; 11 The Boston Consulting Group, Boston, MA 02210, USA; 12 Centre d’Investigations Cliniques (INSERM CIC 1427), Université de Paris, Hôpital Saint-Louis, AP-HP, 75010 Paris, France

**Keywords:** polycythemia vera, thromboembolic events, machine learning, real-world evidence, hydroxyurea, ruxolitinib, biomarkers

## Abstract

Patients with polycythemia vera (PV) are at significant risk of thromboembolic events (TE). The PV-AIM study used the Optum^®^ de-identified Electronic Health Record dataset and machine learning to identify markers of TE in a real-world population. Data for 82,960 patients with PV were extracted: 3852 patients were treated with hydroxyurea (HU) only, while 130 patients were treated with HU and then changed to ruxolitinib (HU-ruxolitinib). For HU-alone patients, the annualized incidence rates (IR; per 100 patients) decreased from 8.7 (before HU) to 5.6 (during HU) but increased markedly to 10.5 (continuing HU). Whereas for HU-ruxolitinib patients, the IR decreased from 10.8 (before HU) to 8.4 (during HU) and was maintained at 8.3 (after switching to ruxolitinib). To better understand markers associated with TE risk, we built a machine-learning model for HU-alone patients and validated it using an independent dataset. The model identified lymphocyte percentage (LYP), neutrophil percentage (NEP), and red cell distribution width (RDW) as key markers of TE risk, and optimal thresholds for these markers were established, from which a decision tree was derived. Using these widely used laboratory markers, the decision tree could be used to identify patients at high risk for TE, facilitate treatment decisions, and optimize patient management.

## 1. Introduction

Polycythemia vera (PV) is a chronic myeloproliferative neoplasm characterized by erythrocytosis and driven, in almost all cases, by mutations in the JAK2 gene [1]. Patients with PV experience a variety of symptoms and signs, including but not limited to pruritus, fatigue, and splenomegaly, and are at increased risk of thrombotic events [2,3]. Thromboembolic events (TE) are a major cause of morbidity and mortality in patients with PV; therefore, treatment strategies aim not only to improve PV-related symptoms but also to prevent or manage thrombotic complications [4,5,6,7].

Risk stratification in PV is designed to estimate the likelihood of TE and includes two risk categories based on age and prior history of thrombosis: high-risk (≥ 60 years old and/or with a history of thrombosis) and low-risk (< 60 years of age with no history of thrombosis) [4]. Higher hematocrit (> 45%; Hct) and high white blood cell counts in patients with PV are associated with an increased risk of thrombosis, and Hct control has been associated with a reduction in thrombotic risk in patients with PV [8,9]. Therefore, Hct control and aspirin use are the current standards of care in all patients with PV to mitigate thrombotic risk. Additionally, high-risk patients with PV require cytoreductive therapy [7].

Hydroxyurea (HU) is a commonly used first-line cytoreductive therapy for high-risk patients with PV [4]. However, even with HU therapy and phlebotomy, adequate Hct control cannot always be sustained, and patients are still at risk of TE [10,11]. Data from the Spanish Registry of PV showed that patients with PV receiving HU had a projected 5- and 10-year probability of thrombosis of 10% and 16%, respectively [10]. Furthermore, treatment with HU has been associated with intolerance and resistance in 10–15% of patients [10,11]. Ruxolitinib is a potent, first-in-class inhibitor of JAK1/JAK2 for the treatment of adult patients with PV who have an inadequate response to or are intolerant of HU and has been shown to reduce the occurrence and risk of thrombosis [12,13,14], improve patients’ quality of life, and improve patient-reported outcomes [15].

Identifying patients with PV at risk of TE and potential markers of TE risk would support the effective therapeutic management of individuals. Machine learning techniques have been used to support the focus of a differential diagnosis, the selection of therapy, and the generation of risk predictions and are increasingly being applied to different areas of hematology, including the management of hematological malignancies [16,17]. The “Polycythemia Vera Advanced Integrated Models for the Prediction of Thromboembolic Events” (PV-AIM) study aimed to utilize the Optum^®^ de-identified Electronic Health Record (EHR) dataset, a large database in the United States, to investigate the incidence of TEs in patients with PV treated with HU and those who switched to ruxolitinib and apply machine learning techniques to identify individuals at risk of TE and potential markers of TE risk. Ultimately, this study aims to provide physicians with the tools to support the effective therapeutic management of individuals with PV and the potential need for timely or proactive change in therapy to reduce a patient’s long-term risk of TE.

## 2. Materials and Methods

### 2.1. Study Design

PV-AIM is an analytical, descriptive, non-interventional, retrospective cohort study of patients with PV using data from the Optum^®^ EHR database (see Supplementary Methods for data source). Patients who were ≥ 18 years of age with a diagnosis of PV and who had received HU were eligible for inclusion in the overall analysis population. However, patients were excluded if they had received fewer than two prescriptions for HU or ruxolitinib, had a diagnosis of myelofibrosis or essential thrombocythemia (ET), and had received other cytoreductive treatment (such as interferon alpha and busulfan).

Patient data describing demographics, history of TE events, history of phlebotomy, clinical observations, laboratory outcomes, and anticoagulant/antiplatelet use were extracted from the Optum^®^ EHR database (Table 1).

**Figure 1:**
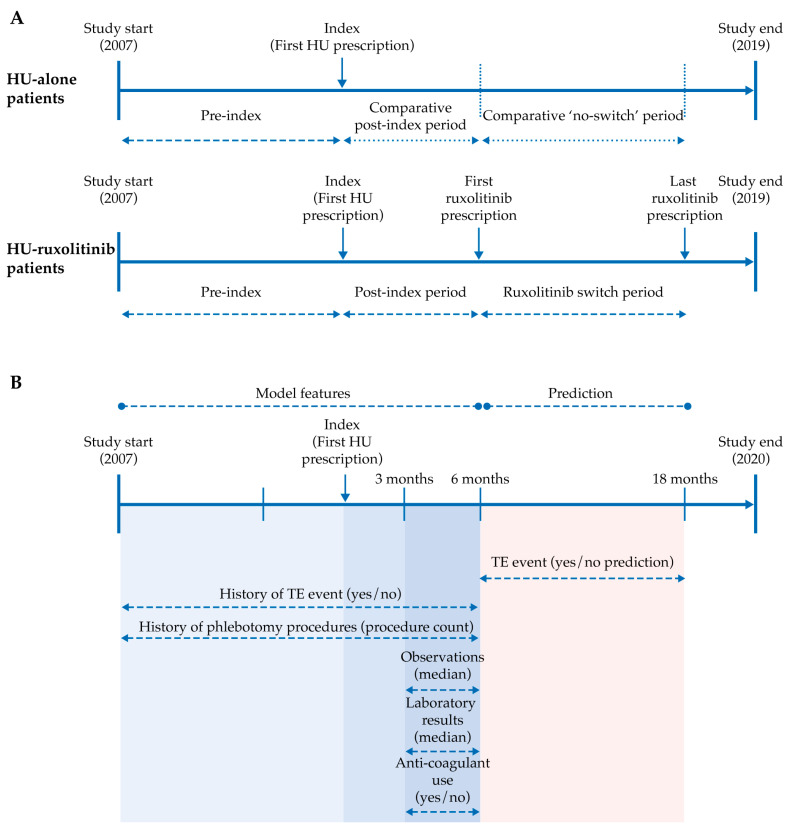
Study designs (**A**) to assess the annual standardized IR of TE in patients with PV treated with HU and then switched to ruxolitinib (HU-ruxolitinib) vs. patients that were treated with HU (HU-alone); (**B**) prediction of TE in patients receiving HU using machine learning techniques. Overall study and patient identification periods extended from 1 January 2007 to 31 December 2019 inclusive. To avoid selection bias when comparing TE incidence in HU-alone and HU-ruxolitinib cohorts, only patients treated with HU up to a cutoff date of the end of December 2013, one year prior to ruxolitinib availability, were included in the HU-alone cohort (**A**). Pre-index period for the determination of annualized IR of TE (**A**) was 365 days. Index date, first date HU-alone or HU-ruxolitinib patients were prescribed HU; pre-index, time from the beginning of the patient’s EHR record to the index date; post-index, time period after the index date, for HU-ruxolitinib, time from the first HU prescription until date of first ruxolitinib prescription, and for HU-alone, time from the first HU prescription until X number of days post-index, where X is the median HU treatment time for the HU-ruxolitinib cohort; ruxolitinib-switch period, time from the first ruxolitinib prescription until the date of last ruxolitinib prescription; HU-alone no switch period, time from the end of ‘post-index period’ until X number of days after, where X is the median ruxolitinib treatment time for the HU-ruxolitinib cohort. HU = hydroxyurea; IR = incidence rate; PV = polycythemia vera; TE = thromboembolic event.

The overall study period (1 January 2007 to 31 December 2019) included pre- and post-index periods, where the index was the first date of HU prescription/administration. Study designs for the analyses are shown in Figure 1.

The initial objective was the evaluation of the incidence rate (IR) in patients treated with HU (HU-alone) and those who changed to ruxolitinib after HU treatment (HU-ruxolitinib), which led to the key objectives that focused on using machine learning techniques to predict the occurrence of TE in patients treated with HU in the extensive and diverse Optum^®^ EHR dataset, considering patients’ clinical, laboratory and therapeutic variables, and to identify novel interactions between patient variables that may act as potential drivers or markers for TE.

### 2.2. Ethics

All Optum^®^ EHR patient data were de-identified and, therefore, Institutional Review Board/Ethics Committee approval was not required. Approval from the Ethical Committee of the General Hospital of Sibenik-Knin County, Sibenik, Croatia was received (22 December 2020) to use patient data from an independent PV registry in Croatia (Reference number 01-22812/1-20); due to the retrospective design of the study, patient consent was waived by the Ethics Committee for this registry and was not required for Optum^®^ EHR patient data. See Supplementary Methods.

### 2.3. Annual Standardized Incidence Rate of TE in Patients with PV Treated with HU-Alone vs. HU-Ruxolitinib

Patients in the HU-alone and HU-ruxolitinib groups were matched using propensity score matching, which accounted for the treatment duration and demographics of patients using the RMatchIt package (MatchIt_3.0.1; https://cran.r-project.org/web/packages/MatchIt/index.html accessed on 21 June 2023) [18]. TE were identified from the International Classification of Diseases-Clinical Modification (ICD-CM) diagnosis codes, and the annualized IR of TE was calculated per 100 patients for the periods pre-index, post-index, and after HU-ruxolitinib switch/no switch (see Supplementary Methods). The full study design is shown in Figure 1A.

### 2.4. Prediction of TE in Patients with PV Receiving HU Using Machine Learning

A random survival forest (RSF) model was constructed using the demographic, clinical and laboratory data extracted from the Optum^®^ EHR database (Table 1) for patients in the HU-alone group who had received at least 6 months of HU treatment, with 18 months of follow-up and at least one laboratory test result and one clinical observation available from 3 to 6 months post-index. The target period for predicting TE was 6 to 18 months post-index (Figure 1B). The model’s performance was assessed using Receiver Operating Characteristic Curve-Area Under the Curve (ROC-AUC). See Supplementary Methods and Supplementary Figure S1 for further details on model development.

Based on the multiple patient variables included in the model (Table 1), the inbuilt RSF variable importance metric was applied to identify those variables with the greatest impact on the prediction of TE; the importance of each variable was based on the degradation of the model’s performance when different variables were removed from the model. Interactions between the top ten most influential variables were assessed for risk of TE in all patients and in patients with/without a history of TE, using the log-rank test. A synergy score was calculated for each interaction (a more significant association with TE than expected), and any synergistic interactions were investigated further.

To investigate patients’ risk of TE, pairs of variables were assessed for the “best split” based on the significance of their interactions; the significance (*p* value) of these two-variable splits was measured by log-rank and generated four groups, or quadrants, from the combinations of “high” and “low” groups for both variables. Rather than assessing a single split threshold using the medians of the two variables only, multiple thresholds were assessed, from which a matrix of *p* values was generated. These matrices were visualized as “heatmaps,” such that regions of significance could easily be identified. The most significant points in this “risk landscape” were identified, and based on these outcomes, clinical “decision trees” were developed.

### 2.5. External Validation of the Model Using an Independent Croatian Dataset

The RSF predictive model was validated using an independent database from Croatia that included retrospective patient data from three community hospitals dating from 26 April 2001 to 11 September 2019 (General Hospital of Sibenik-Knin County, “Dr. Josip Benčević” General Hospital Slavonski Brod, and General Hospital Zadar, Croatia).

Eligible patients were aged ≥ 18 years, had a diagnosis of PV (ICD-10 nomenclature), and had been treated with HU (PV diagnosis reassessed according to World Health Organization criteria for patients diagnosed before 2016) [19]. Key variables identified from the Optum^®^ EHR database were assessed in relation to thrombosis-free survival (TFS) in patients with and without a prior TE history. See Supplementary Methods for additional information.

### 2.6. Statistical Analysis

Absolute values, yes/no or median data were extracted from Optum^®^ EHR database patient information for analysis. Probability curves were compared using Kaplan–Meier plots and log-rank tests, and variable interactions were assessed by a log-rank test (significance *p* < 0.05 for all presented analyses). For analysis of the Optum^®^ EHR database the ranger (version 0.13.1; https://cran.r-project.org/web/packages/ranger/index.html accessed on 21 June 2023) and R (version 4.02; R Foundation for Statistical Computing, Vienna, Austria. https://www.R-project.org/ accessed on 21 June 2023) packages were used for survival and statistical analysis (see Supplementary Methods). For the Croatian database, statistical analyses were performed with MedCalc Statistical Software^®^ (version 19.7, Ostend, Belgium).

## 3. Results

### 3.1. Cohort Selection and Patient Characteristics

**Figure 2:**
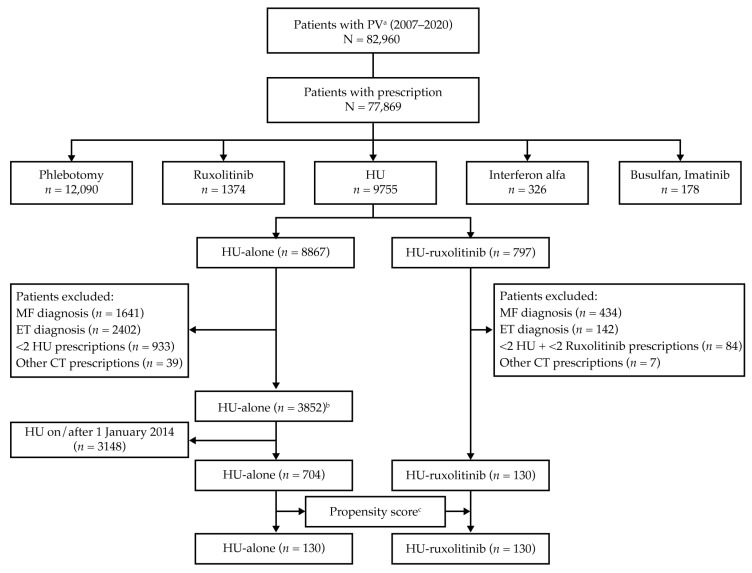
Patient disposition from the Optum^®^ EHR database to the analysis populations. ^a^ PV is defined as ICD-10-CM code D45 or ICD-9-CM code 238.4; ^b^ Of 3852 HU-alone patients, 1012 were used for RSF model development and the prediction of TE, which was based on patients having at least 6 months of HU treatment and 18 months follow-up plus at least one laboratory value during the 3–6 months post-index period; ^c^ Propensity scoring, based on matching patient demographics and treatment period lengths (total treatment time, gender, race, age at index, and region), was applied to align the HU-alone and HU-ruxolitinib cohorts for analysis of annualized IR of TE. HU-alone represents patients who received HU but no ruxolitinib, and HU-ruxolitinib is patients who received HU and changed to ruxolitinib. CT = cytoreductive; ET = essential thrombocythemia; HU = hydroxyurea; IR = incidence rate; MF = myelofibrosis; RSF = random survival forest.

In the extensive Optum^®^ EHR database, 82,960 patients had a diagnosis of PV (median record length of 8.4 years). Of these, 3852 HU-alone patients and 130 HU-ruxolitinib patients were eligible for analysis (Figure 2). For IR analysis, 704 of the 3852 HU-alone patients had received all their HU prescriptions by the cutoff date (December 2013) and, therefore, 130 of these patients were matched to the 130 HU-ruxolitinib patients. See Supplementary Table S1 for matched cohort characteristics.

For RSF model development and prediction of TE, 1012 of the 3852 HU-alone patients were eligible for inclusion (Figure 2). Patient characteristics for the RSF model development and Croatian validation (*n* = 100) cohorts were similar (Table 2).

### 3.2. Annual Standardized IR of TE in PV Patients Treated with HU-Alone vs. HU-Ruxolitinib

**Figure 3:**
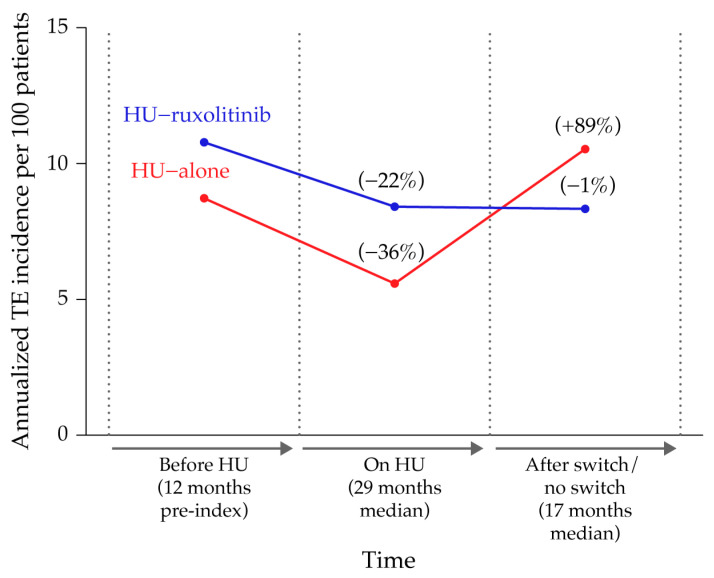
Annualized incidence of TE observed in HU-alone and HU-ruxolitinib patients before HU treatment, during HU treatment and during the HU-ruxolitinib switch period. HU-alone represents patients who received HU but no ruxolitinib, and HU-ruxolitinib is patients who received HU and changed to ruxolitinib. HU = hydroxyurea; TE = thromboembolic event.

Before treatment, the baseline annualized IR of TE per 100 patients in the HU-alone and HU-ruxolitinib cohorts were 8.7 and 10.8, respectively. During the initial period of HU treatment, the IRs decreased to 5.6 and 8.4 in the HU-alone and HU-ruxolitinib cohorts, respectively. In patients who subsequently switched to ruxolitinib (HU-ruxolitinib), the IR remained stable at 8.3; however, for those who continued HU (HU-alone), the IR appeared to rebound and increased markedly to 10.5 over the switch/no switch period (Figure 3).

### 3.3. Prediction of TE in Patients Receiving HU

During model development, it was established that patients who had laboratory and clinical observations collected within the 3–6-month post-index window were at significantly higher risk of TE than patients without these assessments (*p* = 4.6 × 10^−4^); physicians may have considered these patients sufficiently at risk of TE to warrant these assessments (Supplementary Figure S2). The final RSF model achieved a ROC-AUC of > 0.8 for the prediction of TE during the 6- to 18-month post-index period, which demonstrates the strong predictive power of the model (Supplementary Figure S3).

Of all the patient variables analyzed from the Optum^®^ EHR database, ten clinical and laboratory variables were ranked as having the most impact on the prediction of TE (Table 3). As expected, the history of TE was the most influential variable overall, with a >2-fold higher impact score than the other variables; the remaining variables, including anticoagulant and antiplatelet use, had similar impact scores. Notably, neutrophil percentage (NEP), white blood cell count (WBC; × 10^9^/L), lymphocyte percentage (LYP), and red cell distribution width (RDW; %) were the laboratory variables of the greatest importance for predicting TE (Table 3). For LYP, in particular, a significant difference was observed between patients with and without a history of TE (*p* = 7.6 × 10^−3^; Supplementary Figure S4).

**Figure 4:**
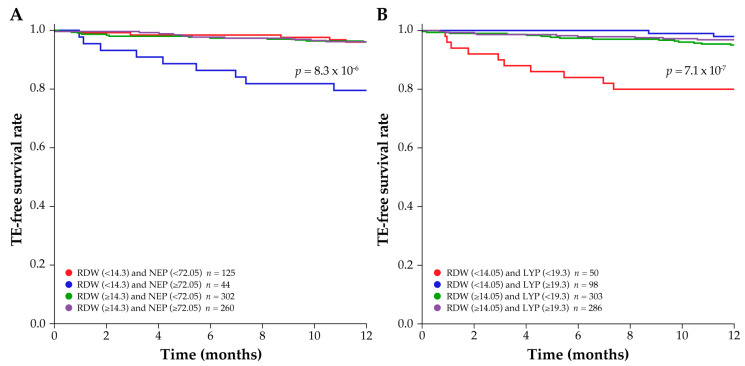
TE-free survival rate over time in patients without a history of TE, based on the median values for the top two synergistic variable pairs: (**A**) NEP and RDW, and (**B**) LYP and RDW. The *p* values (by log-rank) denote significant differences between the synergistic pairs, RDW < 14.3 and NEP ≥ 72.05 group (panel **A**) or the RDW < 14.05 and LYP < 19.3 group (panel **B**) and the other threshold groups investigated for the same two variables. NEP, LYP and RDW are %. LYP = lymphocyte percentage; NEP = neutrophil percentage; RDW = red cell distribution width; TE = thromboembolic event.

**Figure 5:**
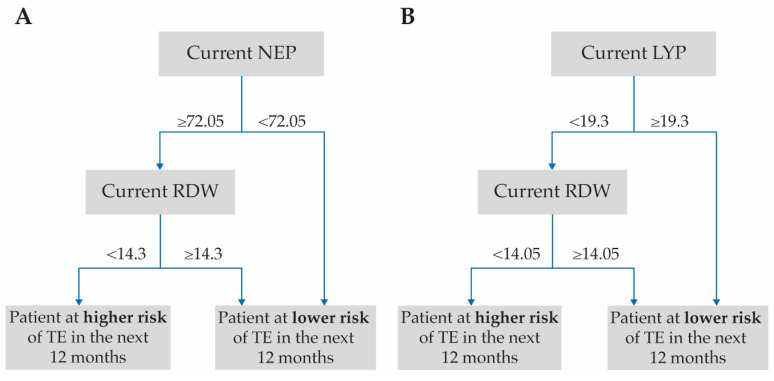
Clinical decision trees developed for the synergistic variable pairs in patients without a history of TE: (**A**) NEP and RDW, and (**B**) LYP and RDW. The thresholds are those identified as significant for these synergistic variable pairs (see Figure 4A,B) and are based on laboratory values taken 3–6 months post-index for patients receiving HU. NEP, LYP and RDW are %. HU = hydroxyurea; LYP = lymphocyte percentage; NEP = neutrophil percentage; RDW = red cell distribution width; TE = thromboembolic event.

Interactions between all the top ten variables were investigated further in all patients, and in patients with and without a history of TE. Analysis saw low synergy scores for the majority of these interactions, such as those for anticoagulant/antiplatelet use, BMI, weight, diastolic blood pressure, and white blood cell count (Table 4), and were not investigated further. However, notable synergistic interactions were observed between the laboratory variables NEP and RDW, and LYP and RDW (Table 4) in patients without any history of TE. Supplementary Figure S5 shows a novel heatmap of the subsequent multiple interactions between RDW and LYP used to determine the best pairwise split associated with TE risk. For patients with no history of TE, the calculated optimal threshold values for higher risk of developing TEs within 12 months were RDW < 14.3 and NEP ≥ 72.05 (%; Figure 4A), and RDW < 14.05 and LYP < 19.3 (%; Figure 4B). Figure 5 shows the final clinical decision trees developed to assess an individual patient’s risk of developing TE while on HU therapy based on their RDW, NEP, and LYP values.

### 3.4. Independent Validation of the RSF Model

**Figure 6:**
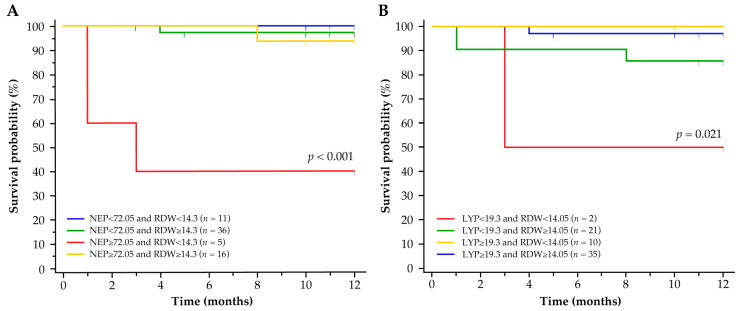
Risk of TE in patients with PV without a history of TE from the independent database in Croatia according to the synergistic variable pairs: (**A**) NEP and RDW, and (**B**) LYP and RDW. *p* values (by log-rank) denote significant differences between the threshold groups. NEP, LYP and RDW are %. LYP = lymphocyte percentage; NEP = neutrophil percentage; PV = polycythemia vera; RDW = red cell distribution width; TE = thromboembolic event.

Consistent with the predictions from the Optum^®^ EHR dataset, the variables NEP, LYP and RDW correctly identified patients without TE history at increased risk of TE in the Croatian database. The optimal thresholds (%) of NEP ≥ 72.05 and RDW < 14.3, and LYP < 19.3 and RDW < 14.05 in the Croatian dataset were predictive of inferior TFS outcomes in patients with no history of TE (Figure 6A and Figure 6B, respectively).

## 4. Discussion

Patients with PV remain at risk of TE, often despite attempts to reduce their risk with first-line treatments such as HU and phlebotomy [10,11]. As such, the focus of the PV-AIM study was to combine machine learning techniques with real-world data from a database representative of the US population to investigate TE risk in patients with PV and identify clinically relevant markers of TE risk. The early identification of potentially “at-risk” patients undergoing treatment with HU, particularly those who do not have a history of TE, may lead to a focused and individual approach to the therapeutic management of patients with PV by physicians, which could improve patients’ outcomes. To our knowledge, PV-AIM is the first in-depth, machine-learning-driven study to identify markers for TE risk in HU-treated patients, which ultimately may support the identification of “at-risk” patients in the clinic.

The IRs of patients with PV in this study were nominally higher than those observed in a meta-analysis of 3236 patients with PV receiving HU [20], which may be a consequence of the marked difference in patient numbers and demographics of the populations in these studies. Patients who receive HU are considered at high risk of TE, and although HU has demonstrated significant efficacy in preventing arterial thromboses, doubts remain as to its ability to prevent recurrent venous thromboembolism [21]. In the PV-AIM study, although patients initially received some protection from TE with HU, this effect was not sustained, and an apparent rebound effect was evident over time, confirming reports that the HU protective effect is not maintained in all patients and that HU-treated patients are still at risk of TE [10]. This apparent escape from the initial protective effects of HU may be partly a consequence of patients losing their responsiveness over time and becoming resistant to HU [10,11]. In contrast, the TE risk remained stable for patients who changed to ruxolitinib. Genomic analysis has suggested that patients with mutations in *TP53* may rapidly develop resistance to HU, whereas high rates of thrombosis and disease progression were noted in patients with *JAK2* homozygous mutations [22]. We cannot speculate on the genetic disposition of patients in this PV-AIM analysis, and although the cohorts in this IR analysis were matched for total treatment time, gender, race, age at index, and region by propensity scoring, other potential differences may have influenced these IR observations; but whether social, financial, health, or genetic differences exist is beyond the scope of this study. However, the observations from this PV-AIM analysis are consistent with clinical observations in the RESPONSE trials, in which a significantly higher percentage of ruxolitinib-treated patients achieved Hct control compared with the best available treatment (62% vs. 19%, respectively; *p* < 0.0001 in RESPONSE 2) [13,23] and a lower rate of TE was observed for up to 5 years (1.2 vs. 8.2 per 100 patient-years, respectively, at 5 years) [24]. Indeed, two meta-analyses support these clinical observations: significantly lower rates of thrombosis were reported in patients with MF and PV treated with ruxolitinib [risk ratio 0.45, 95% confidence interval (CI) 0.23–0.88] [14] and an IR ratio of 0.56 (95% CI, 0.28–1.11) in favor of ruxolitinib versus the best available therapy was observed in patients with PV [12]. Similarly, in a retrospective real-world analysis of patients resistant or intolerant to HU, those who received ruxolitinib had a significantly lower rate of arterial thrombosis compared with patients on the best available treatment (0.4% vs. 2.3%; *p* = 0.03) [25]. This retrospective analysis is supported by the recent randomized, phase II MAJIC-PV study, in which TFS and event-free survival (major thrombosis, hemorrhage, transformation, and death) were significantly improved (*p* = 0.05 and *p* = 0.03, respectively), and Hct was lower with ruxolitinib versus the best available treatment in patients resistant or intolerant to HU [26]. A large prognostic study is underway to confirm these findings (Ruxolitinib versus hydroxycarbamide or interferon as first-line therapy in high-risk polycythemia vera [MITHRIDATE]; https://clinicaltrials.gov/ct2/show/NCT04116502 accessed on 21 June 2023). Collectively, observations from these different studies highlight the benefits of ruxolitinib on TE risk in patients with PV. For patients potentially at risk of TE while receiving HU, a change in therapy may be beneficial.

The PV-AIM study utilized the wealth of patient information available in the Optum^®^ EHR database and novel machine learning techniques to thoroughly analyze patient demographics, history, clinical observations, and laboratory outcomes and to identify the key pre-treatment factors most predictive of TE in patients on HU treatment. In this analysis, the model exhibited strong predictive power and identified notable synergistic associations between the pairs RDW and NEP, and RDW and LYP in patients without a history of TE, as well as the optimal thresholds for patients at low and high risk of TE. Leukocytes may have a causative effect in the initiation of thrombosis, with leukocytosis increasing the risk for thrombosis in patients with PV and ET [27]. LYP, however, expresses the overall change in lymphocytes with regards to inflammation and the immune state (i.e., the ratio of lymphocytes to leukocytes) and, as an inflammatory marker, has been shown to be an independent predictor of lung cancer risk [28]. In our analysis, patients at “higher TE risk” were those with lower LYP values (LYP < 19.3), which is consistent with other reports in patients with PV, where low lymphocyte counts have been associated with worse TFS [29] and the occurrence of venous thrombosis [30]. The association between TE risk and white blood cell counts and threshold values has been investigated [31,32], and the absolute neutrophil count [33] and the combination of LYP and NEP as the neutrophil-to-lymphocyte ratio (NLR) [30] have been reported to be independent risk factors for venous thrombosis but not arterial thrombosis. A high absolute neutrophil count had a negative impact on venous TFS in patients with PV [33], which supports our finding that a higher NEP (≥ 72.05) was predictive of patients at high risk of TE. Although not investigated in our analysis, the higher NLR values of ≥ 5 that resulted in a doubling of the risk for venous thrombosis [30] are consistent with the high neutrophil and low lymphocyte values observed in our study. Interestingly, given the impact of differential white blood cells on TE, this may, in part, explain the stabilizing effect on IR seen with ruxolitinib, which has anti-inflammatory qualities targeting several elements in the adaptive and innate immune systems [34].

Of note was our finding that RDW is a significant factor in predicting TE occurrence. In patients with PV, high RDW has been associated with an increased risk of venous thrombosis [35] and poor TFS [36], and it has been suggested that higher RDW might represent different pathophysiological processes in different patients with PV and ET; however, higher RDW was associated with PV, cardiovascular risk, history of thrombosis, and the need for cytoreductive treatment and is considered a good prognostic marker [36]. In contrast, however, lower RDW (< 14.3%) was associated with an increased risk of thrombosis in PV-AIM, which is consistent with a large single-center study of patients with PV in China in which RDW < 14.5% at diagnosis was associated with worse TFS in high-risk patients with PV, especially for arterial thrombosis, and in patients 50 years of age or with prior thrombosis [29]. There appears to be an inverse relationship between RDW and erythrocyte turnover or clearance, such that a reduction in turnover rate allows older, smaller erythrocytes to remain in circulation, expanding the overall volume and, consequently, the RDW, which may compensate for changes in erythropoiesis [37]. Thus, increased RDW may suggest stressed erythropoiesis, whereas decreased RDW may suggest increased erythropoiesis despite cytoreduction. When comparing these studies, it should be noted that reports may include baseline RDW, patients with ET, and thrombosis and death as a combined endpoint [36], whereas PV-AIM assessed RDW in patients with PV during HU treatment and, therefore, may suggest that erythropoiesis increased despite treatment with HU.

The synergistic variable pairs identified in PV-AIM have greater predictive potential than individual variables or other combinations and are of particular value in patients who would be considered at low risk for TE, based on their age and history of TE alone at the start of HU therapy. Collectively, the outcomes from PV-AIM and other studies [29,30,36] highlight the value of routinely assessed laboratory variables, and it is postulated that proactive inclusion of laboratory variables such as NEP, LYP, and RDW may improve the identification of patients at low and high risk of thrombosis. Importantly, both “low risk” and “high risk” patients should be monitored routinely during HU therapy to gauge patients’ continued risk of TE. Likewise, consideration of cardiovascular risk factors, such as hypertension, would be beneficial when assessing TE risk in patients with PV, given that cardiovascular risk factors are strongly linked with TE occurrence, TFS, and survival [38,39], and, therefore, different subgroups of patients considered at low risk for TE might be at risk [39]. The optimal thresholds for LYP, NEP, and RDW formed the basis for the two decision trees constructed to guide physicians in categorizing patients without a history of TE as high or low risk for developing TE within 6 to 18 months of starting HU treatment, and to support physicians’ decisions in proactively monitoring and reassessing therapy options in a timely manner to reduce potential TE risk. Following the development of the decision trees, the predictive model was validated to determine its reproducibility in different populations. Remarkably, the NEP, LYP, and RDW patterns identified from the Optum^®^ EHR database could be applied to the independent Croatia PV population, and these combinations of NEP, LYP, and RDW were able to correctly identify the patients with PV in the real-life community setting that were at increased risk of future TE, which supports the broad applicability of these findings to real-world data and registries beyond the USA.

As expected, given the observational and retrospective nature of this analysis and the use of real-world data, we acknowledge some limitations to this analysis. The period from which patient data was extracted was prolonged, and physician treatment practices may have changed over this period; however, sufficient patient numbers were needed, and the required pre- and post-index periods were accommodated to ensure the quality and completeness of the dataset. Although data for a substantial number of patients with PV was available within the Optum^®^ EHR database, strict inclusion and exclusion criteria were required to obtain a focused cohort of patients for the machine learning analysis, which substantially reduced the number of eligible patients. As such, this focused analysis population may have excluded some patients of interest that may have influenced the risk of TE, such as those on different anticoagulants or antiplatelet therapies. The Optum^®^ EHR database includes routinely collected clinical data from a wide range of sources (physician offices, emergency rooms, laboratories, and hospitals); therefore, data may have been entered differently at the source with the possibility of missing, invalid, unrecorded, or unknown data, inaccuracies, and/or technical errors, but also possibly as a consequence of a subjective medical judgment of diagnosis, drug, and/or procedural codes. In addition, medication use may have been overestimated as there are no guarantees of patients being dispensed their medication or using their medication as prescribed. As such, HU treatment may be different between the HU-alone and HU-ruxolitinib groups. Despite these potential limitations, the outcomes from this analysis were validated externally through the Croatian database, which corroborated the overall PV-AIM findings.

The identification of easy-to-determine laboratory markers that are predictive for TE risk in patients with PV and the development of clinically applicable decision trees present an exciting new opportunity for physicians to identify patients who do not have a history of TE but are potentially at risk of TE and would benefit from closer surveillance and follow-up. RDW, NEP, and LYP are routine laboratory parameters and, therefore, are inexpensive and practical tools in the clinic. Ultimately, early identification of “at-risk” patients and close monitoring during treatment provide a comprehensive and personalized approach to patient management, which may promote timely changes in treatment to prevent a major cause of morbidity and mortality. Machine learning techniques have proved to be a useful tool in this study, and further studies are now needed to refine the risk for arterial or venous thrombosis.

## Figures and Tables

**Table 1:** Patient data extracted from the Optum^®^ EHR database.

Feature	Patient Data Extracted from the Optum^®^ EHR Database
Demographics	Age at index, gender, race, ethnicity, region, division
History	Thromboembolic event (TE) history ^a^, number of phlebolotomy procedures
Laboratory values ^b^	Hematocrit (Hct), white blood cell count (WBC), platelet count (Plt), red blood cell distribution width (RDW), lymphocyte percentage (LYP), hemoglobin (HGB), neutrophil percentage (NEP)
Anticoagulant/ antiplatelet drugs used/prescribed ^a^	Apixaban, Rivaroxaban, Edoxaban, Dabigatran warfarin, UFH (unfractioned heparins), LMWH (low molecular weight heparins, e.g., Enoxaparin, Nadroparin), Fondaparinux, Acetylsalicylic acid, Ticlopidine, Clopidogrel, Radugrel (prasugrel), Cangrelor, Abciximab (anti GpIIb/IIIa), Abciximab
Observations ^b^	Respiratory (RSP), heart rate (HRT), pulse (PLS), weight (WGT), height (HGT), body mass index (BMI), systolic blood pressure (SBP), diastolic blood pressure (DBP), alcohol ^a^, smoking ^a^

^a^ Patients categorized as yes or no; ^b^ Median data extracted. History of TE and phlebotomy procedures were taken from the beginning of the patients’ records until 6 months post-index and all clinical and laboratory data were collected during the 3- to 6-month post-index (See Figure 1A) window.

**Table 2:** Patient characteristics of patients with PV in the Optum^®^ EHR and Croatian databases used to develop and validate the final RSF model.

Characteristics	Optum^®^ EHR Dataset (*n* = 1012)	Croatian Dataset (*n* = 100)
Females (%)	51	45
Age (years), median (IQR)	73 (64–80)	65 (56–72)
*JAK2-V617F* mutation, *n*	-	100
Palpable splenomegaly (%)	-	23
History of thrombosis (%)	16.1	32
Arterial hypertension (%)	68.8	79
Diabetes mellitus (%)	13.7	16
Hyperlipidemia (%)	15.9	16
Smoking (%)	12.8	17
Anticoagulant or antiplatelet use ^a^ (%)	48 (3 to 6 months)/ 93 (anytime)	81 (anytime)
Leukocytes (×10^9^/L), median (IQR)	7.7 (5.9–10.3)	7.2 (6.0–11.7)
Granulocytes (×10^9^/L), median (IQR)	-	3.8 (2.8–5.4)
Neutrophils (%), median (IQR)	70 (62–78)	68 (60–78)
Lymphocytes (%), median (IQR)	19.5 (13.0–26.3)	22 (14.8–30)
Platelets (×10^9^/L), median (IQR)	278 (203–381)	269 (200–454)
Hematocrit (%), median (IQR)	43 (39.7–46.3)	42 (40–46)
Hemoglobin (g/L), median (IQR)	140 (130–151)	144 (129–154)
RDW (%), median (IQR)	17.0 (14.5–19.3)	16.0 (14.3–17.5)

The Optum^®^ EHR patient dataset was used to build the RSF model to predict TE at 6 to 18 months after the first HU treatment. The Croatian dataset was used to validate the RSF predictive model. ^a^ For the Croatian dataset, the only anticoagulant/antiplatelet used during the study were acetylsalicylic acid and warfarin; for the Optum^®^ EHR dataset see Table 1. See Supplementary Methods for additional information. IQR = interquartile range; RDW = red cell distribution width; RSF = random survival forest; TE = thromboembolic event.

**Table 3:** Top 10 most influential observational and laboratory variables for the prediction of TE in rank order of impact.

Rank	Variable Name	Score
1	TE history (yes/no)	0.16
2	Median BMI	0.08
3	Median DBP	0.065
4	Median weight	0.064
5	Median NEP	0.059
6	Median WBC	0.059
7	Median LYP	0.058
8	Use of anticoagulant/antiplatelet therapy	0.058
9	Age at index	0.054
10	Median RDW	0.053

BMI = body mass index; DBP = diastolic blood pressure; LYP = lymphocyte percentage; NEP = neutrophil percentage; RDW = red cell distribution width; TE = thromboembolic event; WBC = white blood cell count.

**Table 4:** Synergistic combinations of the top ten most influential model variables.

Interaction		Cohort	Synergy ^a^		
Variable 1	Variable 2		Expected *p* Value	Observed *p* Value	Score
NEP	RDW	Without TE history	1.80 × 10^−3^	8.30 × 10^−6^	223.11
LYP	RDW	Without TE history	1.30 × 10^−4^	7.10 × 10^−7^	177.27
DBP	Weight	Without TE history	3.50 × 10^−3^	4.30 × 10^−4^	8.16
Weight	RDW	Without TE history	4.10 × 10^−4^	8.40 × 10^−5^	4.92
LYP	RDW	All	9.70 × 10^−6^	2.10 × 10^−6^	4.70
BMI	Anticoagulant/ antiplatelet	Without TE history	7.60 × 10^−5^	1.70 × 10^−5^	4.43
WBC	RDW	With TE history	1.40 × 10^−2^	4.80 × 10^−3^	2.88
BMI	RDW	All	1.90 × 10^−3^	7.00 × 10^−4^	2.69

^a^ Synergy score is defined as the product of the individual log-rank significances of variables 1 and 2 (expected) divided by the log-rank significance of the two-variable model (observed). BMI = body mass index; DBP = diastolic blood pressure; LYP = lymphocyte percentage; NEP = neutrophil percentage; RDW = red cell distribution width; TE = thromboembolic event; WBC = white blood cell count.

## Data Availability

Data for this study was made available through a third-party data use agreement from Optum, a commercial data provider in the US. Further release of the dataset is not possible due to this data use agreement. The data from the Croatian dataset are not publicly available due to privacy and ethical restrictions, but data sharing may be considered upon reasonable request directed to Ivan Krečak.

## Supplementary Materials

The following supporting information can be downloaded at: https://www.mdpi.com/article/10.3390/biomedicines11071925/s1, Methods; Statistical analysis; Figure S1: Prediction of TE using machine learning through RSF model from HU-alone patient data (*n* = 1012); Figure S2: TE-free survival in patients with laboratory and clinical observations taken within the 3- to 6-month post-index window and in patients without these data; Figure S3: Evaluation of the RSF model for the prediction of TE (6 to 18-months post index) for an unseen cohort (holdout set); Figure S4: Boxplot showing the difference in median LYP in patients with and without a history of TE; Figure S5: Heatmaps showing the risk landscape of all possible combinations of median LYP and RDW values for: (A) All patients, (B) Patients with a history of TE, and (C) Patients without any history of TE; Table S1: Patient characteristics for the matched HU-alone and HU-ruxolitinib cohorts from the Optum^®^ EHR database.
