# Supplementary material for: Identifying Patients with Polycythemia Vera at Risk of Thrombosis after Hydroxyurea Initiation: The Polycythemia Vera—Advanced Integrated Models (PV-AIM) Project
Source: Biomedicines. 2023 Jul 7;11(7):1925. doi: 10.3390/biomedicines11071925 (PMC10377437; doi:10.3390/biomedicines11071925)
Supplement: Supplementary file 1 [file biomedicines-11-01925-s001.zip › biomedicines-2348444-supplementary.pdf]

Supplementary Material

Methods.

Optum® EHR and Croatian datasets

The Optum® de-identified Electronic Health Record (EHR) dataset contains electronic medical records for 105 million patients currently (2007–2020), with at least ten million patients in each of the main geographical regions (West, Midwest, Northeast, and South) in the USA, and with proportions of age, gender, ethnicity, and race that are similar to the overall US population. The Optum® EHR database includes the electronic medical records from 82,960 patients with polycythemia vera (PV) with a median record length of 8.4 years. Furthermore, it includes information relating to diagnosis (in particular for PV, other myeloproliferative neoplasms and associated thrombotic events), demographics, treatment administration and prescription (in particular for hydroxyurea [HU] and ruxolitinib), procedures, laboratory tests, and signs and symptoms. These data can come from physician offices, emergency rooms, laboratories, and hospitals, and can provide information from clinical and inpatient stays.

Data for this study were retrieved from the Optum® EHR dataset (2007–2019), which contains de-identified and aggregated clinical and medical administrative data from at least 65 US healthcare delivery organizations across 50 states. The database includes information from more than 150,000 providers, 7000 clinics and 2000 hospitals. The participating healthcare delivery organizations provide data captured by their local EHR systems. For longitudinal analysis of patients, the average number of follow-up years for patients in the database ranges from ≥ 1 year (~66%) to ≥ 5 years (~38%).

Annual standardized incidence rate of thromboembolic events (TE) in patients with PV treated with HU-alone vs HU-ruxolitinib

Propensity score matching was done using RMatchIt package (MatchIt_3.0.1), and the score matching was run with respect to total treatment time, gender, race, age at index, region with the "nearest" algorithm, and ratio = 1.

The HU treatment period was determined from the HU-ruxolitinib cohort as the period from the index date (first HU prescription) until the first prescription of ruxolitinib. The median HU treatment period was then calculated (it was equal to 876 days) and applied to the HU-alone cohort (index plus 876 days as the comparative post-index period). Similarly, the median ruxolitinib exposure duration ("switch period") in the HU-ruxolitinib cohort (it was equal to 510 days) was used to calculate the comparative "no-switch" period in the HU-alone cohort. Annualized IR was calculated as below:

$$Anualized\ Incidence\ Rate = \left(\frac{No.\ Patients\ with\ Events}{Sum\ (Patients\ Days)} \times 365\right) \times 100$$

Prediction of TE in patients with PV receiving HU using machine learning

When multiple measurements were available, the median value was taken for continuous variables. Demographics held by in the Optum® EHR also included: age at index, gender, race, ethnicity, region, and division. The event to be predicted or target variable (dependent variable) was the occurrence of a TE in the 6 to 18 months after index along with days to a TE (from 6 months post index).

The model features (independent variables) consisted of history of TE (yes/no), history of phlebotomy (number of procedures carried out) from the beginning of the patients' record until 6 months post index, clinical observations (respiratory, heart rate, pulse, weight, height, body mass index, systolic blood pressure [SDP], diastolic blood pressure [DBP]), hematology laboratory results (hematocrit [Hct], white blood cell count [WBC], platelet counts, red blood cell distribution width [RDW], lymphocyte counts, neutrophil percentage [NEP], and hemoglobin [HGB]) and anticoagulant/antiplatelet use/prescription (yes/no) were all collected in the 3 to 6 months window after index.

External validation using independent Croatian dataset

Clinical (age, sex, presence of palpable splenomegaly, history of TE, and anticoagulant use) and laboratory variables (hemoglobin, Hct, WBC, absolute granulocyte counts, absolute lymphocyte counts and platelet counts, RDW, LYP and NEP) were collected in the 3- to 6-month window after index date. The index date was defined as the time of first HU prescription.

Indications for HU treatment in patients without TE history were age > 60 years (*n* = 40/68, 58.8%), vasomotor disturbances (*n* = 10/68, 14.7%), pruritus, night sweats and fatigue (*n* = 10/68, 14.7%), abdominal discomfort due to splenomegaly (*n* = 6/68, 8.8%), and iron deficiency-related symptoms (*n* = 2/68, 2.9%).

Thrombosis-free survival time was calculated to predict the risk of TE 6 to 18 months after an index date with failure being an arterial or venous TE. TEs were defined individually for every patient through medical chart review. Arterial TE were defined as myocardial infarction, transitory cerebral ischemic attack, acute cerebral ischemic stroke, or acute peripheral arterial occlusion, whereas venous TE were defined as peripheral vein thrombosis, pulmonary embolism, or splanchnic vein thrombosis.

Time-to-TE event probability curves were compared using Kaplan-Meier plots and log rank tests. Statistical analyses were performed with MedCalc Statistical Software® (version 19.7, Ostend, Belgium) and significant *p* values were set at < 0.050 for all presented analyses.

Cardiovascular risk factor analysis

Optum® EHR dataset

Arterial hypertension was ≥ 140 SBP and/or ≥ 90 DBP. Smokers were defined as those that answered "currently smoking" in an observational survey within 12 months of first HU treatment. Patients with these CV risk factors were included in the PV-AIM model.

Croatian dataset

The definition of arterial hypertension in the Croatian dataset was "at baseline" (at disease diagnosis) and included ≥ 140 SBP and/or ≥ 90 DBP and/or the use of antihypertensives at disease diagnosis. Hyperlipidemia was defined as the use of hypolipidemics and/or total cholesterol ≥ 200 mg/dL and/or low-density lipoprotein (LDL) ≥ 70 mg/dL. All patients included in the study with diabetes were previously evaluated by an endocrinologist and the presence of diabetes was defined as the use of antidiabetic drugs and/or fasting glucose ≥ 126 mg/dL and/or 2h oral glucose tolerance test (OGTT) ≥ 200 mg/dL and/or HbA1c 6.5% and/or random glucose sample ≥ 200 mg/dL with the presence of signs and symptoms that may be attributed to diabetes. Smokers were defined as "active smokers". This patient population with these CV risk factors was used to validate the PV-AIM model.

Statistical analysis

Model

A random survival forest (RSF) model was chosen due to its wide use and acceptance, robustness, tendency not to over-fit training data and amenability to explanation via inherent variable importance score. Random forest can also be run with time-to-event time data in the form of survival forests by replacing the traditional information/impurity score with statistical tests, such as log-rank or C-index, when constructing the trees in the forest.

We used the ranger R package to implement our random forest model due to its parallelisation and its ability to produce probability scores as a form of prediction confidence, which can be harnessed during model evaluation. We used the training set/test set approach 70/30 and receiver operating characteristic curve-area under the curve (ROC-AUC) analysis to evaluate the performance of our model. Random forest's inherent variable/feature importance was used to aid model explanation.

Pairwise variable/feature interactions

Due to the base classifier being a decision tree, which is built by sequentially splitting variables, random forest based variable importance score is influenced by variable interactions (e.g., where a variable may only be important in combination with another variable). In this section, we attempt to elucidate these interactions amongst the top variables.

Here we developed a method to further explore the pairwise interactions across variables in the context of time-to-event analysis. For the continuous variables in the top ten variables that were selected by variable importance ranking, we carried out an exhaustive search of all possible pairwise combinations across all possible variable values. Each combination was evaluated via the log-rank metric, and the best pairwise splits were returned for each variable pair. We also created a record of this exhaustive search, which allowed us to map the risk landscape given two variables and a defined cohort of patients.

To investigate cases of extreme synergy, instances in which two variables split the given cohort into high-risk and low-risk patients were far better than either variable alone. We used a simple synergy scoring metric to rank variable in terms of synergy (S): Sab = (Pa * Pb)/Pab where, for a given patient cohort, Pa and Pb were the maximum possible (log-rank derived) *p* values for variable "a" and variable "b" and Pab was the maximum *p* value possible from the combination of variables "a" and "b". This synergy was intended to capture variable that may provide exclusive non-redundant information when attempting to split a cohort based on risk and may provide an added insight into the functional/clinical rationale of a model.

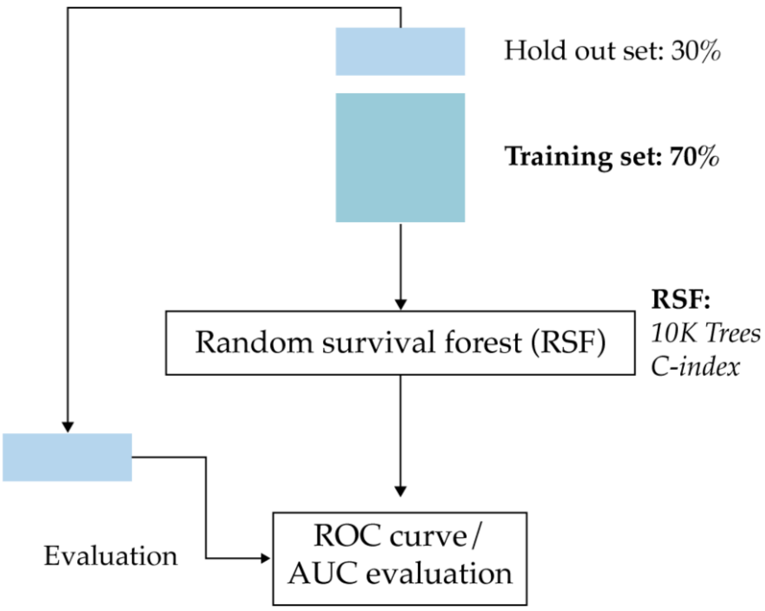

Thirty percent of the data was withheld from the model training. The RSF was trained on 10K trees with a C-index split function. The RSF model was then used to predict TEs in this unseen 'holdout' cohort. The TE predictions were evaluated against the known labels using AUC of the generated ROC curve, which is indicative of the model's performance.

AUC = area under the curve; HU = hydroxyurea; ROC = receiver operating characteristic; RSF = random survival forest; TE = thromboembolic event.

Figure S1. Prediction of TE using machine learning through RSF model from HU alone patient data (*n* = 1,012).

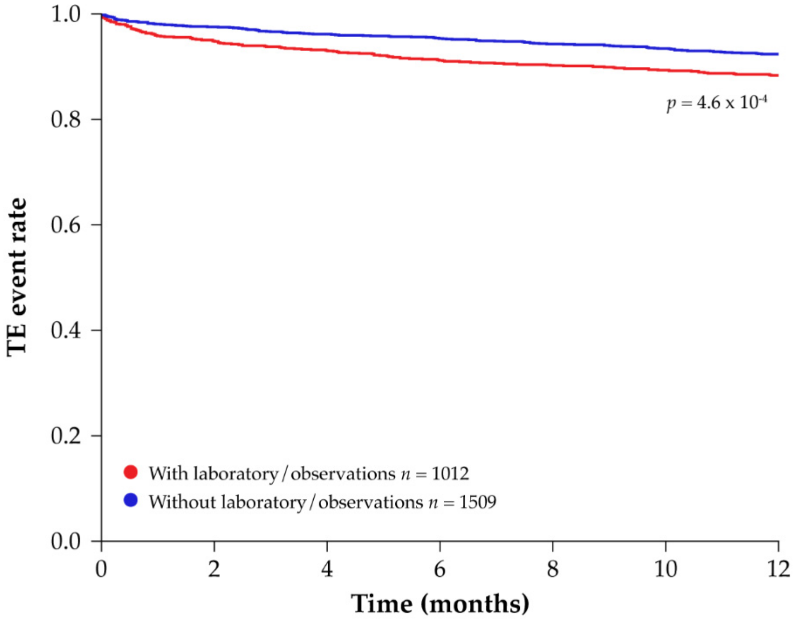

P value represents the difference in TE-free survival in patients with ≥1 laboratory and ≥1 clinical observation taken within the 3 to 6-months post-index window compared with patients without these data.

TE = thromboembolic event.

Figure S2. TE-free survival in patients with laboratory and clinical observations taken within the 3 to 6-month post-index window and in patients without these data.

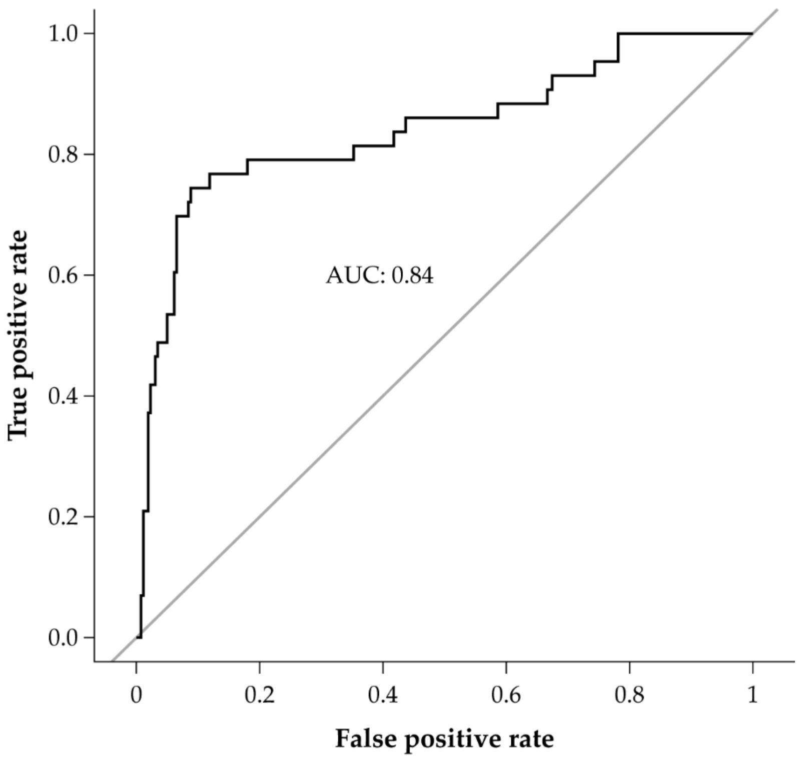

ROC-AUC analysis was used to evaluate the model's performance. ROC-AUC values nearing 1 indicate good predictive capabilities.

AUC = area under the curve; ROC = receiver operating characteristic (curve); RSF = random survival forest; TE = thromboembolic event.

Figure S3. Evaluation of the RSF model for the prediction of TE (6 to 18-months post index) for an unseen cohort (holdout set).

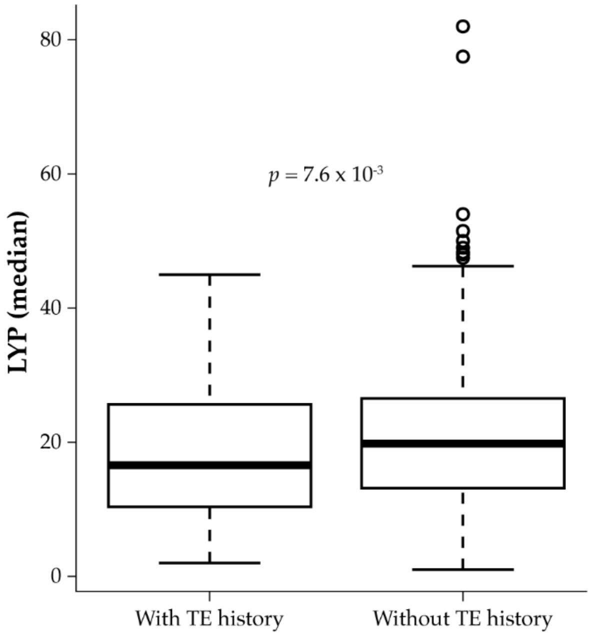

P value denotes the difference between median LYP for patients with and without a history of TE.

LYP = lymphocyte percentage; TE = thromboembolic event.

Figure S4. Boxplot showing the difference in median LYP in patients with and without a history of TE.

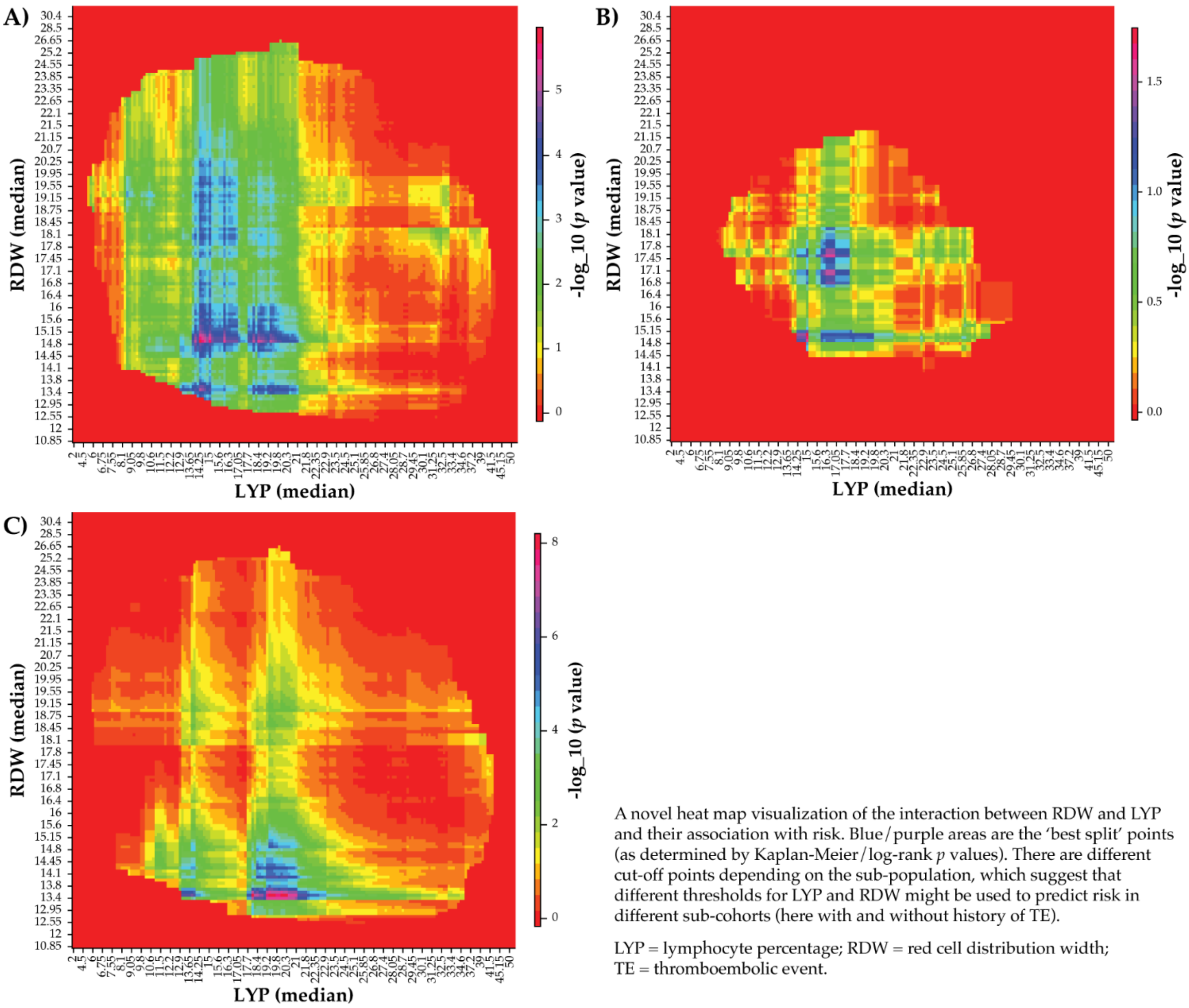

A novel heat map visualization of the interaction between RDW and LYP and their association with risk. Blue/purple areas are the 'best split' points (as determined by Kaplan-Meier/log-rank *p* values). There are different cut-off points depending on the sub-population, which suggest that different thresholds for LYP and RDW might be used to predict risk in different sub-cohorts (here with and without history of TE).

LYP = lymphocyte percentage; RDW = red cell distribution width; TE = thromboembolic event.

Figure S5. Heatmaps showing the risk landscape of all possible combinations of median LYP and RDW values for: A) All patients, B) Patients with a history of TE, and C) Patients without any history of TE.

Table S1. Patient characteristics for the matched HU-alone and HU-ruxolitinib cohorts from the Optum® EHR database.

Characteristic	HU-alone (*n* = 130)	HU-ruxolitinib (*n* = 130)
Mean age at index, years	68.4	67.9
Total treatment period		
Mean, days	1255.7	1776.7
Median, days	1354	1629
Gender, proportion		
Male	0.58	0.58
Female	0.42	0.42
Race, proportion		
Caucasian	0.95	0.95
Unknown/other	0.03	0.03
US geographical division, proportion		
East South Central	0.02	0.06
Middle Atlantic	0.09	0.13
Mountain	0.05	0.04
New England	0.07	0.04
Pacific	0.00	0.02
South Atlantic/West South Central	0.23	0.25
West North Central	0.22	0.18
Other/Unknown	0.02	0.02

HU = hydroxyurea.
